# Prediction of Recombination Spots Using Novel Hybrid Feature Extraction Method via Deep Learning Approach

**DOI:** 10.3389/fgene.2020.539227

**Published:** 2020-09-17

**Authors:** Fatima Khan, Mukhtaj Khan, Nadeem Iqbal, Salman Khan, Dost Muhammad Khan, Abbas Khan, Dong-Qing Wei

**Affiliations:** ^1^Department of Computer Science, Abdul Wali Khan University Mardan, Mardan, Pakistan; ^2^Department of Statistics, Abdul Wali Khan University Mardan, Mardan, Pakistan; ^3^Department of Bioinformatics and Biological Statistics, School of Life Sciences and Biotechnology, Shanghai Jiao Tong University, Shanghai, China; ^4^State Key Laboratory of Microbial Metabolism, Shanghai-Islamabad-Belgrade Joint Innovation Center on Antibacterial Resistances, Joint Laboratory of International Cooperation in Metabolic and Developmental Sciences, School of Life Sciences and Biotechnology, Shanghai Jiao Tong University, Ministry of Education, Shanghai, China; ^5^Peng Cheng Laboratory, Shenzhen, China

**Keywords:** DNA sequence, feature selection, deep neural network, classification, system biology, novel feature extraction

## Abstract

Meiotic recombination is the driving force of evolutionary development and an important source of genetic variation. The meiotic recombination does not take place randomly in a chromosome but occurs in some regions of the chromosome. A region in chromosomes with higher rate of meiotic recombination events are considered as hotspots and a region where frequencies of the recombination events are lower are called coldspots. Prediction of meiotic recombination spots provides useful information about the basic functionality of inheritance and genome diversity. This study proposes an intelligent computational predictor called iRSpots-DNN for the identification of recombination spots. The proposed predictor is based on a novel feature extraction method and an optimized deep neural network (DNN). The DNN was employed as a classification engine whereas, the novel features extraction method was developed to extract meaningful features for the identification of hotspots and coldspots across the yeast genome. Unlike previous algorithms, the proposed feature extraction avoids bias among different selected features and preserved the sequence discriminant properties along with the sequence-structure information simultaneously. This study also considered other effective classifiers named support vector machine (SVM), K-nearest neighbor (KNN), and random forest (RF) to predict recombination spots. Experimental results on a benchmark dataset with 10-fold cross-validation showed that iRSpots-DNN achieved the highest accuracy, i.e., 95.81%. Additionally, the performance of the proposed iRSpots-DNN is significantly better than the existing predictors on a benchmark dataset. The relevant benchmark dataset and source code are freely available at: https://github.com/Fatima-Khan12/iRspot_DNN/tree/master/iRspot_DNN.

## Introduction

Meiotic recombination is the process of exchanging alleles between homologous chromosomes, which take place during meiosis (Lichten and Goldman, 1995; Petes, 2001; Liu et al., 2012, 2016). It is a vital biological process that is carried out in two phases; meiosis and recombination. In meiosis, the genome is divided into two equivalent parts which are known as daughter cells that take part in the production of a new living organism. While in the recombination process, the different gametes are joined to make new genetics combinations (Kabir and Hayat, 2016). It is essential for cell division and an important process to make heredity variances (Paul et al., 2016; Zhang and Kong, 2018b). Hence, the meiotic recombination gives opportunities for natural exchange of hereditary variations (Chen et al., 2013; Zhang and Kong, 2018b), which causes the genome to create more hereditary differences and speed up the genetic progress. The meiotic recombination does not take place randomly in a chromosome but occurs in some regions of a chromosome. In general, the region that exhibits a high frequency of recombination is considered as hotspots, whereas the region that exhibits low frequency of recombination is considered as coldspots (Liu et al., 2012; Dong et al., 2016). The study of recombination spots provides useful information about the basic functionality of inheritance and genome diversity. Additionally, it gives valuable insights about variation in DNA sequence and patterns, which may help to know the position of alleles that cause different diseases (Abeysinghe et al., 2003; Hey, 2004).

Owning to the importance of meiotic recombination, several predictors have been introduced in the literature using machine learning methods for identification of recombination spots (Zhou et al., 2006; Jiang et al., 2007b; Liu et al., 2012, 2016, 2017a; Chen et al., 2013; Li et al., 2014; Qiu et al., 2014; Dong et al., 2016; Kabir and Hayat, 2016; Wang et al., 2016; Dwivedi, 2018). For example, Liu et al. (2012) proposed a model for discrimination of recombination spots using an increment of diversity with quadratic discriminant analysis (IDQD) method and k-mer approach. Jiang et al. (2007b) proposed RF-DYMH predictor based on gapped dinucleotide composition (GDC) technique for feature formulation and RF as a classification algorithm. Chen et al. (2013) proposed iRSpot-PseDNC based on “pseudo dinucleotide composition” (PseDNC) with SVM. The authors employed PseDNC with physiochemical properties for feature extraction and SVM as a classification engine. Liu et al. (2016) proposed iRSpot-DACC based on dinucleotide-based autocross covariance (DACC) with SVM as a learning algorithm. The DACC incorporated global sequence order information along with local DNA properties to construct a feature vector. Liu et al. (2017a) proposed iRSpot-EL model for discrimination of recombination spots. The proposed model applied PseKNC and DACC along with ensemble classifier. Kabir and Hayat (2016) proposed iRSpot-GAEnsC using different sequence formulation methods, such as nucleotide, di-nucleotide, and tri-nucleotide along with ensemble classifiers. Qiu et al. (2014) proposed iRSpot-TNCPseAAC that combined TNC and PseAAC (pseudo amino acid composition) techniques to formulate DNA samples. The TNC method was used to integrate DNA local or short-range sequence order information, whereas, the PseAAC method was applied to integrate DNA global and long-range sequence order information. Zhang and Kong (2018b) proposed iRSpot-PDI using PseAAC as a feature extraction technique along with the BFS (best first search) method for feature selection. Maruf and Shatabda (2018) proposed iRSpot-SF computational model for the identification of hotspot using a sequence based feature method with SVM. The author used different K-mer composition approaches to extract optimum features. Zhang and Kong (2018a) proposed iRSpot-ADPM using di-nucleotide composition (DNC) as a sequence formulation technique and SVM as a classification engine. Jani et al. (2018) proposed iRecSpot-EF for the classification of recombination hotspots and coldspots. The authors employed K-mer for feature extraction, AdaBoost technique for feature selection, and logistic regression as classification algorithms. The methods mentioned above have applied single layer conventional machine learning methods that are unable to discriminate hotspots and coldspots accurately.

Recently, Khan et al. (2019b) proposed iRSpot-SPI for predictions of hotspots and coldspots based on multilayer deep learning algorithm. The proposed model used sequence-structure information along with deep learning as a discriminative method. The proposed model achieved the highest accuracy; however, the authors ignored optimization (i.e., tuning) of hyper-parameters of the DNN model. We argue that with un-tuning parameters, a model can generate unstable results, which affect the overall performance of the model. In short, the existing models are summarized in Table 1 according to applied feature extraction methods and machine learning algorithms.

**Table 1:** Summery of exiting model according to feature extraction methods and machine learning algorithms.

**Model name**	**Classification algorithm**	**Feature extraction method**
RF-DYMHC (Jiang et al., 2007b)	RF	GDC
IDQD (Liu et al., 2012)	IDQD	K-mer
iRSpot-PseDNC (Chen et al., 2013)	SVM	PseDNC
iRSpot-TNCPseAAC (Qiu et al., 2014)	SVM	TNC and PseAAC
iRSpot-GAEnsc (Kabir and Hayat, 2016)	KNN, SVM, RF	DNC and TNC
iRSpot-DACC (Liu et al., 2016)	SVM	DACC
iRSpot-EL (Liu et al., 2017a)	SVM	DACC and PseKNC
iRSpot-ADPM (Zhang and Kong, 2018a)	SVM	DNC
iRSpot-SF (He et al., 2018)	SVM	K-mer Composition, TF-IDF, gapped k-mer composition, and reverse complement k-mer composition (RCC)
iRecSpot-EF (Jani et al., 2018)	Logistic regression	RCC
iRSpot-SPI (Khan et al., 2019b)	DNN	GDC, RCC, and PseTNC

**Figure 1:**
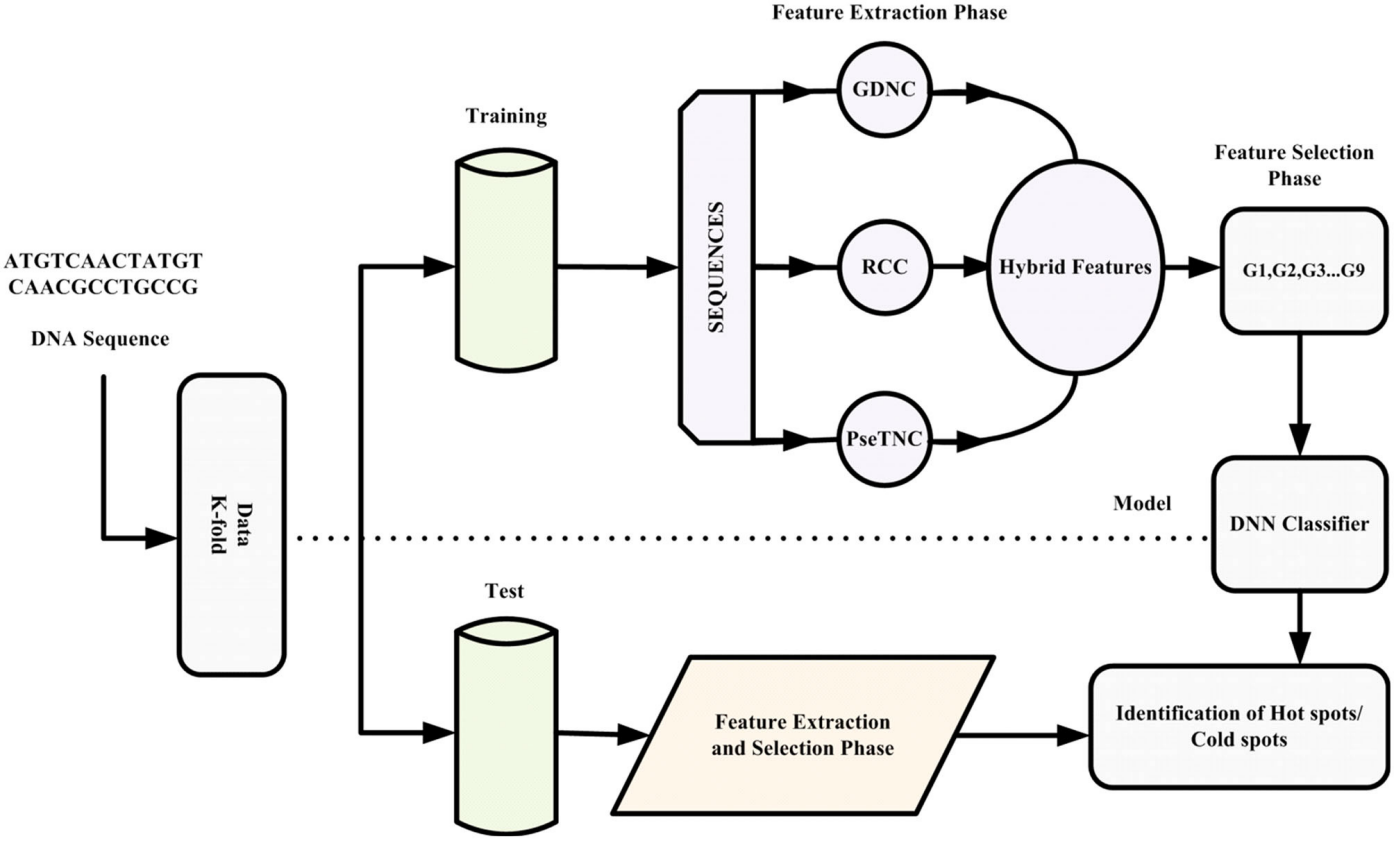
Framework of proposed iRSpot-DNN.

This paper proposes an intelligent computation model based on a novel hybrid feature extraction method along with optimized DNN for the prediction of recombination hotspots and coldspots. Moreover, the proposed model employed SVM-RFE (Guyon et al., 2002; Zhang et al., 2006) for discriminant feature selection. The proposed model was designed to follow Chou's five-steps rule mentioned comprehensively in a series of publications (Chou et al., 2011; He et al., 2015; Jia et al., 2015; Khan et al., 2019a). The framework of the proposed iRSpot-DNN is shown in Figure 1. Firstly, we selected a valid benchmark dataset that contained recombination hotspots and coldspots sequences. The benchmark dataset was split into training and testing dataset. Secondly, different feature extraction methods were employed to construct feature vectors. Thirdly, we obtained discriminant features using feature selection method. Fourthly, we proposed a novel method that considered the contribution of different feature extraction methods in order to avoid biasness and preserved sequence discriminative properties. Fifthly, the proposed model applied a grid search approach for hyper-parameters tuning. Sixthly, the DNN model with optimized hyper-parameters was applied to predict recombination spots. Finally, the performance of the proposed model was evaluated on a selected benchmark dataset using a 10-fold cross-validation test. Based on evaluation results, the iRSpot-DNN yielded the highest success rate of 95.81%, sensitivity of 96.17%, specificity of 95.92%, and Matthews correlation coefficient of 0.915. Furthermore, the outcome of iRSpot-DNN was compared with the current predictors, and the comparison results illustrated that the proposed iRSpot-DNN outperformed the current predictors.

## Method and Material

### Benchmark Dataset

The selection of a consistent and standard benchmark dataset is the first step toward building an intelligent, accurate, and reliable prediction model. In order to build a reliable prediction model, this paper selected a standard benchmark dataset presented in Jiang et al. (2007b), which is used in several papers (Liu et al., 2012; Chen et al., 2013; Qiu et al., 2014; Yang et al., 2018; Zhang and Kong, 2018b; Khan et al., 2019b). We formulated the benchmark dataset as follows:

(1) $$\begin{array}{r} S=S^{+}\cup \;S^{-} \end{array}$$

Here S^**+**^ represents hotspots and S^**-**^ represent coldspots. *U* is the set theory operator representing a union of both the S^+^ and S^−^. Initially, the dataset contained 490 S^+^ sequences and 591 S^−^ sequences. To eliminate redundant and homologous sequences, we applied CD-HIT (Li and Godzik, 2006; Fu et al., 2012) software that removed sequences having similarity more than 75%. The updated dataset contained 478 S^+^ sequences and 572 S^−^ sequences.

### Sequence Formulation Methods

In the previous section, we discussed the construction of a benchmark dataset that contained DNA hotspots and coldspots sequences. In this section, we formulate biological sequences of different lengths in a feature vector with the same length. Since the statistical machine learning models deal only with numerical descriptors of equal length (Chou, 2015; Noi and Kappas, 2018). Therefore, the biological sequences are required to transform (formulate) into a uniform discrete feature vector before they are given to a computation model. However, the biological sequence may lose pattern or order information at the time of the sequence formulation process. Therefore, various methods in the area of computational biology have been proposed for formulation of DNA, RNA and protein sequences into a distinct feature vector with preserved the sequence pattern and order information (Chen et al., 2013; Qiu et al., 2014; Kabir and Hayat, 2016; Liu et al., 2016; Wang et al., 2016; Yang et al., 2018; Zhang and Kong, 2018b). Besides, web servers have been developed that can be used to convert DNA, RNA, and protein sequences into features vectors according to user's need (Liu et al., 2015, 2017b; Chen et al., 2019).

In this paper, firstly, we applied different sequence formulation/feature extraction methods, such as Gapped di-nucleotide composition (GDC), Reverse complement composition (RCC) and PseTri-Nucleotide Composition (PseTNC) to transform biological sequences into feature vectors. Secondly, we derive a novel formula that generated different features groups based on contribution of the selected feature.

Let suppose D is a DNA sequence from dataset S with length L, can be expressed in mathematical form as Equation (2).

(2) $$\begin{array}{r} \text{D}={\text{D}}_{1},{\text{D}}_{2},{\text{D}}_{3},\ldots {\text{D}}_{\text{L}} \end{array}$$

Where D_1_ ϵ {A, C, G, T} and i ϵ (1, 2, 3…L). D_1_ is the nucleotide at first residue position; D_2_ is the nucleotide at second residue position and so no.

#### Gapped Di-nucleotide Composition

Di-nucleotide composition method is widely used for a sequence formulation; however, this method is considered a correlation between two nucleotides having the same properties. In order to consider K intervals correlation, we used GDC that describes the correlation of every two pairs of nucleotides with a total number of K intervals in a sequence. Many research papers have been applied to the GDC (Jiang et al., 2007b; Ghandi et al., 2014; Tang et al., 2016; Maruf and Shatabda, 2018; Khan et al., 2019b) as a feature extraction method. The GDC computes the cumulative frequency of every two pairs of nucleotides with k number of intervals in a sequence. The GDC can be formulated using Equation (3).

(3) $$\begin{array}{r} g_{\left(\kappa \right)}^{𝔦}=\frac{O_{\left(\kappa \right)}^{𝔦}}{n_{\left(\kappa \right)}} \end{array}$$

Where $O_{\left(\kappa \right)}^{𝔦}$ is the number of i^th^ observed in two nucleotides, k is the intervals of bases and *n*_(κ)_ is the entire population size of two nucleotide with k interval bases (Tang et al., 2016).

#### Reverse Complement Composition

The reverse complement of a sequence can be achieved by reversing the letters of a sequence, i.e., exchanging A and T and exchanging C and G. A genome sequence obscures valuable information in a hidden pattern as well as in a reverse complement pattern, that provides most important regularity information (Lopez et al., 1999). Rev(k-mer) composition can be expressed as Equation (4):

(4) $$\begin{array}{r} RC_{\text{Composition}}\left(S_{\text{i}}\right)=\frac{1}{L}Count_{\text{R}}\left(S_{\text{i}}\right),\forall k=3,4,5\ldots \end{array}$$

#### PseTri-Nucleotide Composition

The PseTri-Nucleotide composition (PseTNC) method was introduced by Chou's et al. for a sequence formulation. The PseTNC method considers three nucleotide compositions (NC) during the sequence formulation and also considers preserving sequence order information (Kabir and Yu, 2017; Khan et al., 2020). In PseTNC, the occurrence frequency of three NC can be computed using the method mentioned in Du et al. (2012) and Li et al. (2016). The PseTNC method can be represented in general form with K-tuple as Equation (5).

(5) $$\begin{array}{r} \text{D}={\left[f_{1}^{K-tuple}f_{2}^{K-tuple}\ldots f_{i}^{K-tuple}\ldots f_{4^{k}}^{K-tuple}\right]}^{T} \end{array}$$

$f_{i}^{K-tupple}$ is the normalized frequency of ith k-tuple nucleotide in a DNA sequence. We can observe from Equation (5) that increasing the value of K, increases the dimensionality of the feature vector. In order to limit the feature vector dimension to 64 possible combinations, we re-write Equation (5) in the form of 3-tuple PseTNC as Equation (6):

(6) $$\begin{array}{r} \text{D}={\left[f_{1}^{3-tuple}f_{2}^{3-tuple}\ldots f_{64}^{3-tuple}\right]}^{T} \end{array}$$

Where, $f_{1}^{3-tuple}=f\left(AAA\right)$, $f_{2}^{3-tuple}=f\left(AAC\right)$, … $f_{64}^{3-tuple}=f\left(TTT\right)$

Equation (6) can be written in terms of Equation (2)

(7) $$\begin{array}{r} \text{D}={\left[D_{1}\;D_{2}\ldots D_{64}\;D_{64+1}\ldots D_{64+\lambda }\right]}^{T} \end{array}$$

(8) $$\begin{array}{r} d_{\text{v}}=\{\begin{array}{c} \frac{f_{v}^{3-tuple}}{\sum_{i=1}^{64}f_{i}^{3-tuple}+w\sum_{j=1}^{\lambda }\theta_{j}}\;1\leq v\leq 64\\ \frac{w\theta_{v-64}}{\sum_{i=1}^{64}f_{i}^{3-tuple}+w\sum_{j=1}^{\lambda }\theta_{j}}\;\left(64+1\right)\leq v\leq \left(64+\lambda \right) \end{array} \end{array}$$

In Equation (8) *w* denotes weight factor and θ denotes correlation factor, given as follows:

(9) $$\begin{array}{r} \theta_{j}=\frac{1}{L^{*}-j}\Sigma_{i=1}^{L^{*}-j}\theta \left(T_{i};T_{i+j}\right)j=1,2,\ldots \lambda <L^{*} \end{array}$$

### Discriminant Feature Selection

In the previous section we described different feature extraction/formulation methods that generate various numbers of features as shown in Table 2. Feature vector play a vital role in a model prediction process, however, a feature vector with a high dimension space may negatively effects the outcome of a prediction model due to noisy, redundant, and irrelevant features. A number of techniques have been proposed in the literature to reduce feature vector dimensionality by removing the redundant, noisy and irrelevant features. In this paper we employed SVM-RFE (SVM-Recursive Feature Elimination) (Guyon et al., 2002; Zhang et al., 2006) technique as a feature selection method to reduce dimensionality of a feature vector with minimum loss of discriminative features. As a result we obtained selected features vectors summarized in Table 3. It is to be noted that we employed SVM-RFE method on PseTNC feature vector to eliminate noisy feature and to obtain selected features, however, the selected features could not significantly improved the performance of the model. Hence, we utilized all the generated features, i.e., 66 of the PseTNC in prediction of recombination spots.

**Table 2:** Number of features generated by different feature extraction method.

**Feature extraction method**	**Number of features**
Gapped dinucleotide composition	128
Reverse complement composition	680
PseTNC	66

**Table 3:** Summary of selected features.

**Feature extraction method**	**Number of features**	**Selected features**
Gapped dinucleotide composition	128	5
Reverse complement composition	680	12
PseTNC	66	66

### Hybrid Feature and Feature Selection

In this section, we derived a novel formula based on feature extract methods, such as GDC, RCC, PseTNC, and feature extracted in iRecSpot-EF (Jani et al., 2018) and their contributions. The novel formula can be written as Equation (10).

(10) $$\begin{array}{r} F=a\text{G}+\lambda \left(\text{ R}+\text{PseTNC}\right)+\left(1-\lambda \right)\left(H\right) \end{array}$$

In Equation (10) “a” and “λ” are two parameters having values between (0,1), which represent the contribution of a method in the feature vector. G represents GDC, R represents RCC, PseTNC represents PseTri-Nucleotide Composition and H represents 425 features generated by iRecSpot-EF (Jani et al., 2018). Further, G represents five selected features, R represents 12 selected features and PseTNC represents 66 features (Khan et al., 2019b). The parameters “a” and “λ” are used to measure the contribution of the feature extraction methods in the dimension of a hybrid feature vector. A different combination of “a” and “λ” generates various numbers of features, as shown in Table 4. For example, using a combination of 0 (i.e., “a” = 0 and “λ” = 0), a total of 425 features are generated, which is represented by G1. Similarly, a combination of 0 and 1 (i.e., “a” = 0 and “λ” = 1) generates 78 features, which is represented by G2 and so on. Hence, the number of selected features are based on the values of “a” and “λ.”

**Table 4:** Number of features in each group computed using Equation (10).

**Feature group**	**A**	**λ**	**Total features**
G1	0	0	425
G2	0	1	78
G3	1	0	430
G4	1	1	83
G5	0.5	0	428
G6	0	0.5	252
G7	0.5	0.5	255
G8	0.5	1	81
G9	1	0.5	257

### Classification Algorithms

#### Deep Neural Network

**Figure 2:**
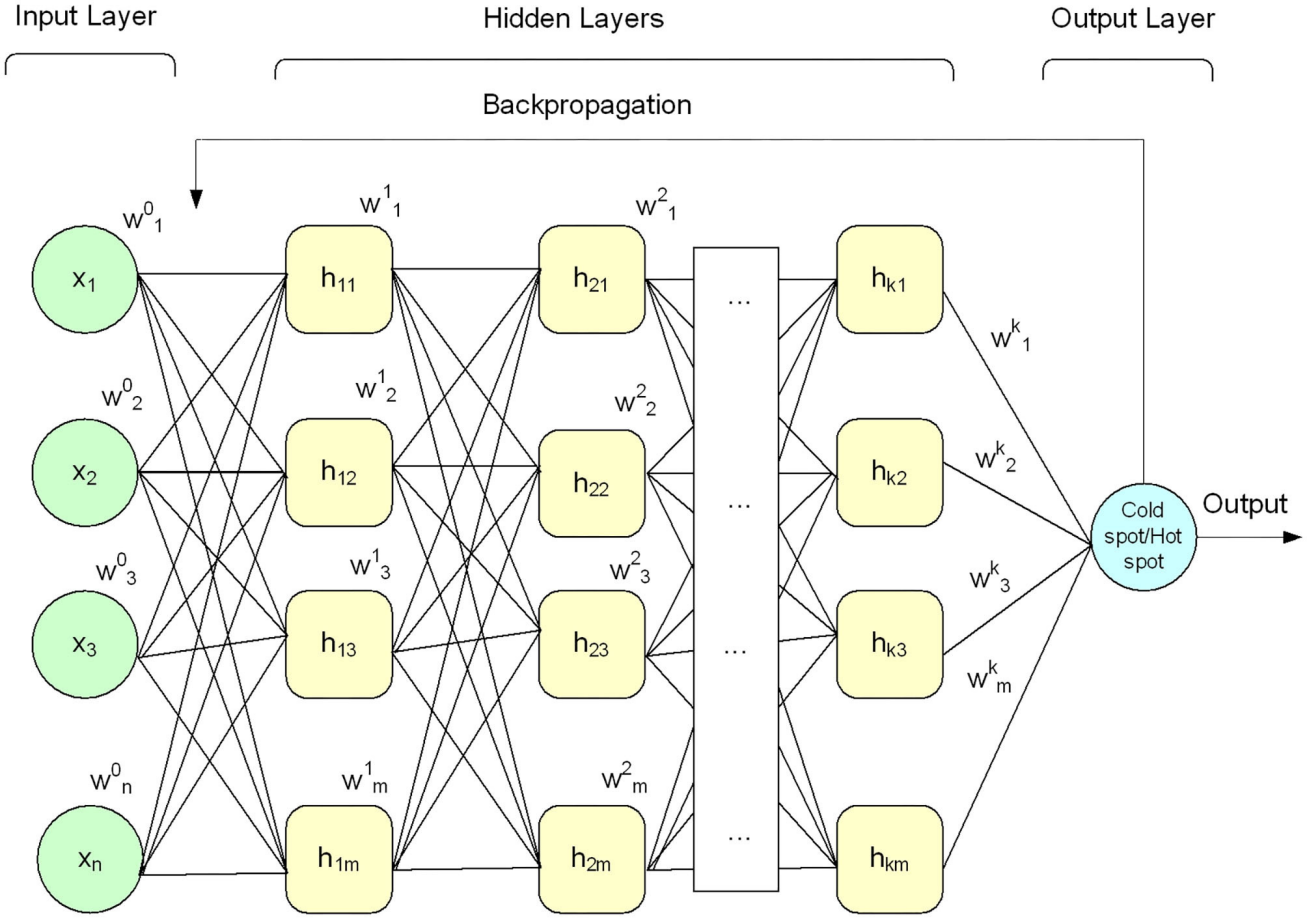
Architecture of deep neural network.

Deep learning algorithms apply neural networks that learn features from the data directly and then make decisions. Recently, deep learning algorithms received significant attention in the field of bioinformatics and computational biology (Lecun et al., 2015; Angermueller et al., 2016; Kelley et al., 2016; Mamoshina et al., 2016; Quang and Xie, 2016; Min et al., 2017; Cohn et al., 2018; Miao and Miao, 2018; Telenti et al., 2018; Zhang et al., 2019). A DNN model comprises an input layer, output layer, and multiple hidden layers, as shown in Figure 2. The given input data is passed through each layer, where the output of the previous layer is presented as input to the next layer.

The performance (i.e., accuracy) of the DNN model depends upon the number of hidden layers in a network. In general, a network configures with a large number of hidden layers in a training or testing phase can lead to excellent learning and ultimately improve the accuracy of the model (Khan et al., 2019a). However, it may lead to major problems, such as the complexity of the model, computation cost, and overfitting (Liu et al., 2015; Chen et al., 2019).

Deep learning methods have been successfully employed in several areas, including speech recognition (Deng et al., 2012; Sainath et al., 2013), image processing (Krizhevsky et al., 2012; Couprie et al., 2013; Tompson et al., 2014), natural language processing (Mikolov et al., 2011; Bordes and Weston, 2014), bio-Engineering (Acharya et al., 2018; Zhu et al., 2019) and genomics (Khan et al., 2019b). In addition, different research papers have proved that the deep learning methods performed better than conventional machine learning techniques used for various complex learning problems (Deng et al., 2012; Leung et al., 2014; Ma et al., 2015). Due to the remarkable performance of the deep neural network in different domains, in this paper, we apply the DNN model as a classifier for the prediction of recombination spots.

In this paper, the DNN model was configured with a small number of hidden layers (i.e., five hidden layers) along with input layer and output layer as shown in Figure 2 to keep the model simple (i.e., less computationally costly) and avoid the model overfitting problem. Each layer of the model was configured with multiple processing nodes (i.e., neurons). Firstly, a given feature vector *X*{*x*_1_*x*_2_*x*_3_…*x*_*n*_} was fed to the input layer and computed output using Equation (11). Secondly, the output of the input layer was fed as input to the first hidden layer and produced a new output. Thirdly, the output of the first hidden layer was provided as input to the second hidden layer and computed output again. This process was continued till we reach to the output layer. The output layer generated binary value, i.e., 0 and 1. The value 0 represents hotspot, and 1 represents coldspots. Furthermore, different activation functions were employed at the input layer and hidden layers, however, the DNN model with hyperbolic tangent (Tanh) activation function generated promising results compared with other activation functions (see Table 6). The softmax function was applied at the output layer of the deep neural network to map the output (non-normalized output) of the last layer to a probability distribution to predict the output class. Moreover, stochastic gradient descent and backpropagation were used to optimize weight and bias value to minimize the error. In addition, we employed regularization and dropped out methods to overcome any possible occurrence of the model overfitting issue. Mathematically, a single layer computation can be expressed as Equation (11).

(11) $$\begin{array}{r} Y=g\left(\overset{n}{\underset{i=1}{\sum }}Xw_{i}^{k}+b_{i}\right) \end{array}$$

Where g represents activation function, X represents feature vector, *n* represents the number of features, k represents the layers, and b represents bias value.

#### Support Vector Machine

Support vector machine (SVM) is an effective supervised machine learning technique mostly used for classification and regression. SVM method was first introduced by Cortes and Vapnik (1995) for binary classification problems; however, later on, it was modified for multiclass problems (Ahmad et al., 2015). SVM converts the input data into a high dimensional features space based on transformation, and then define the best possible separating hyper-plane (Qiu et al., 2014). The key points of SVM are the ability to handle large and noisy datasets while avoiding overfitting (Zavaljevski et al., 2002). SVM algorithm can be applied with different kernels; however, SVM with Gaussian Radial Basis Function (RBF) generally generated promising results. Additionally, the SVM can be configured with two other parameters, i.e., C, used for controlling the cost of misclassifications, and γ, used for handling the non-linear classification (Qian et al., 2015; Ballanti et al., 2016). Further details on SVM and its parameter optimization are given in Chou and Elrod (2002) and Cai et al. (2003).

#### K-nearest Neighbor

K-nearest neighbor (KNN) (or Lazy learning) algorithm is a popular algorithm used for both classification and regression purposes (Hu et al., 2016). However, it is mostly used for classification problems (Ali et al., 2015; Zuo et al., 2015; Khan et al., 2017). KNN is an instance-based and non-parametric learning algorithm and can be considered a simple machine learning algorithm (Donaldson, 1967; Qin et al., 2013). KNN algorithm applies Euclidian distance formula to compute distance amongst the instances for classification. The principal characteristic of the KNN is minimum computation times during the training phase; however, it takes a long time during the testing phase. In KNN algorithm, the value of K plays a significant role and it is used to control the fine-tuning of the algorithm. The model becomes less stable when the value of K decreases, Inversely the model go toward more stability when the value of K increases (Harrison, 2018). The KNN algorithm generates promising performance on the dynamic type of data that changes and updates quickly (Van Der Malsburg, 1986; Kondarasaiah and Ananda, 2004). The KNN algorithm becomes slower when the number of samples or examples increases.

#### Random Forest

Random Forest (RF) was proposed by Breiman (Lou et al., 2014; Sitokonstantinou et al., 2018), is an ensemble learning method. The RF algorithm generates a large number of decision trees, in which every single tree produces classification results and then merged all the results of all decision trees using the voting method to generate the final result (Jiang et al., 2007a; Sitokonstantinou et al., 2018). Feature selection in RF is random, i.e., it is not using all the features; it divides the features into different trees and then merges the final result of every tree (Jiang et al., 2007a). Two parameters that are ntree and mtry are needed to be set up for achieving better accuracy. The ntree is the number of trees, while the mtry is the number of samples/variables in each split (Noi and Kappas, 2018). The RF algorithm produces better performance on a large dataset; however, it experience with overfitting in case the dataset is too noisy.

## Performance Metrics

The performance of a newly constructed predictor based on statistical machine learning algorithms can be evaluated through some procedures before it applies in a real production environment (Baratloo et al., 2015). However, before moving forward, we need to consider the following two questions: (a) what measurement metrics should be adopted to evaluate the performance of a new predictor? (b) what test approach should be employed to compute the measurement metrics? Several metrics have been proposed in the literature for performance evaluation of a machine learning model (Chou, 2001a,b; Xu et al., 2013; Lin et al., 2014; Zhang et al., 2016; Liu et al., 2017c, 2018; Feng et al., 2019; Tahir et al., 2019). In all these metrics, accuracy is considered as the most eminent metric for the performance measurement of a model, however, only the accuracy cannot be sufficient to assess the model significance (Guo et al., 2014; Akbar and Hayat, 2018). Therefore, a set of four different measurement metrics along with the accuracy were considered to evaluate the performance of a predictor. These metrics are: (i) overall accuracy (ACC), (ii) sensitivity (SN), (iii) specificity (SP), and (iv) Mathew's correlation coefficient (MCC). The ACC determines a ratio of number of corrected predictions made by the model to the total number of input samples. The SN returns true positive rate of a model whereas the SP is opposite of the SN and compute true negative rate of a model. The MCC uses all positive and negative instances and produces output in the range of +1 and −1. Further, the details and meanings of these metrics are clearly mentioned in series of publications (e.g., see Chen et al., 2007, 2013; Guo et al., 2008; Qiu et al., 2014; Kabir and Hayat, 2016; Sabooh et al., 2018; Zhang and Kong, 2018b; Khan et al., 2019b; Raza, 2019).

In this paper, we considered the aforementioned four metrics to assess the outcomes of the proposed iRSpot-DNN for the prediction of recombination spots. According to Chou's symbol studying in signal protein peptides (Chou, 2001a), the four metrics can be represented in the following equations in order to make them easily understandable to most experimental scientists.

(12) $$\begin{array}{r} \text{ACC}=1-\;\frac{H_{-}^{+}+\;H_{+}^{-}}{H^{+}+\;H^{-}};\;0\;\leq \text{ ACC }\leq \;1 \end{array}$$

(13) $$\begin{array}{r} \text{SN}=1-\;\frac{H_{-}^{+}}{H^{+}};0\;\leq \text{ SN }\leq \;1 \end{array}$$

(14) $$\begin{array}{r} \text{SP}=1-\;\frac{\;H_{+}^{-}}{\;H^{-}};\;0\;\leq \text{ SP }\leq \;1 \end{array}$$

(15) $$\begin{array}{r} \text{MCC}=\frac{\;1-\;\left(\;\frac{H_{-}^{+}+\;H_{+}^{-}}{H^{+}+\;H^{-}}\;\right)\;}{\sqrt{\;\left(1+\;\frac{H_{+}^{-}-\;H_{-}^{+}}{\;H^{+}}\;\right)\;\left(1+\;\frac{H_{-}^{+}-\;H_{+}^{-}}{\;H^{-}}\;\right)}};\;-1\;\leq \text{ MCC}\leq \;1 \end{array}$$

In the above equations, *H*^+^ represents the total number of hotspots, *H*^−^ represents the number of coldspots. Similarly $H_{-}^{+}$ represents the number of hotspots wrongly predicted as coldspots and $H_{+}^{-}$ represents the number of coldspots that are incorrectly predicted as hotspots.

The next challenge is how to assess the quality of a new predictor using the metrics values. For this purpose, three methods, such as jackknife, independent dataset, and K-fold cross-validation are widely applied in the literature (Chen et al., 2013; Liu et al., 2017a; Yang et al., 2018; Zhang and Kong, 2018a,b; Kong and Zhang, 2019) to examine the performance and robustness of a predictor. It is to be noted that in the cited literature, there is no independent dataset is available so for, that is why we are unable to apply independent dataset method whereas the jackknife method is computationally expensive because of its working mechanism. Hence, In this study, we employed K-fold cross-validation (i.e., K = 10) method as it has been adopted by several investigators to assess the quality of their predictors (Zhou et al., 2006; He et al., 2018; Kong and Zhang, 2019) and comparatively less time consuming technique compare with jackknife method.

## Experimental Results and Discussion

In this section, we discuss the efficiency and significance of the proposed iRSpot-DNN. Firstly, we discuss the hyper-parameter optimization using a grid search technique. Secondly, we assess the performance of the DNN algorithm using various sequence formulation techniques along with the hybrid features. Thirdly, the performance of the DNN is compared with other machine learning algorithms. Finally, the outcome of the proposed iRSpot-DNN is compared with recently published models.

### Hyper-Parameter Optimization

Deep learning algorithms consider a number of hyper-parameters during model configuration. The configurations of these parameters have a significant impact on the performance of a learning algorithm. Unlink regular parameters, the hyper-parameters are specified by user during the model setup. Typically, it is a challenging task to know what value to be configured for the hyper-parameters of a learning model on a given dataset. Therefore, different approaches, i.e., manual trail, grid search (Fowler, 2000), and random search are commonly used for hyper-parameter tuning. The manual trail and random search approaches for hyper-parameter tuning are laborious, time-consuming and un-methodical. Hence, we apply a grid search technique to find optimal hyper-parameters for the proposed model. In order to apply the grid search approach, we build a model for different combinations of hyper-parameters, evaluate the model for every combination and store the results. The set of hyper-parameters that gives the best result amongst all combinations is selected and considered as the optimal parameter set for the proposed model. During the hyper-parameter optimization, we consider the most influential parameters, such as activation function, learning rate, and a number of iterations. We examined the model hyper-parameter on all groups of features using grid search approach, however, the promising results were obtained on G3 group features. The optimal hyper-parameters obtained through the grid search method for the proposed model are presented in Table 5.

**Table 5:** List of hyper-parameters with optimized values.

**S. No**	**Parameter**	**Optimum configuration value**
01	Training iterations	1,000
02	Learning rates	0.1
03	Activation function at output layer	Softmax
04	Activation function at hidden layer	Tanh
05	Seed	6
06	Number of hidden layers	4
07	Number of neuron at hidden layers	430-413-318-251-182-96-2
08	Weight initialization function	XAVIER function
09	Optimizer	SGD method
10	Momentum	0.9
11	Updater	ADAGRAD function

The learning rate in machine learning algorithms is a vital component and determines the step size a model takes at each iteration. The step size is the amount that weights updated during the model training phase. The value of the learning rate can be a small positive value in the range between 0.0 and 1.0. A small value of the learning rate may lead to overfitting and takes a longer time to train the model, whereas a large value can quickly train the model. However, it may ignore some best characteristics of features being used during the model training.

**Figure 3:**
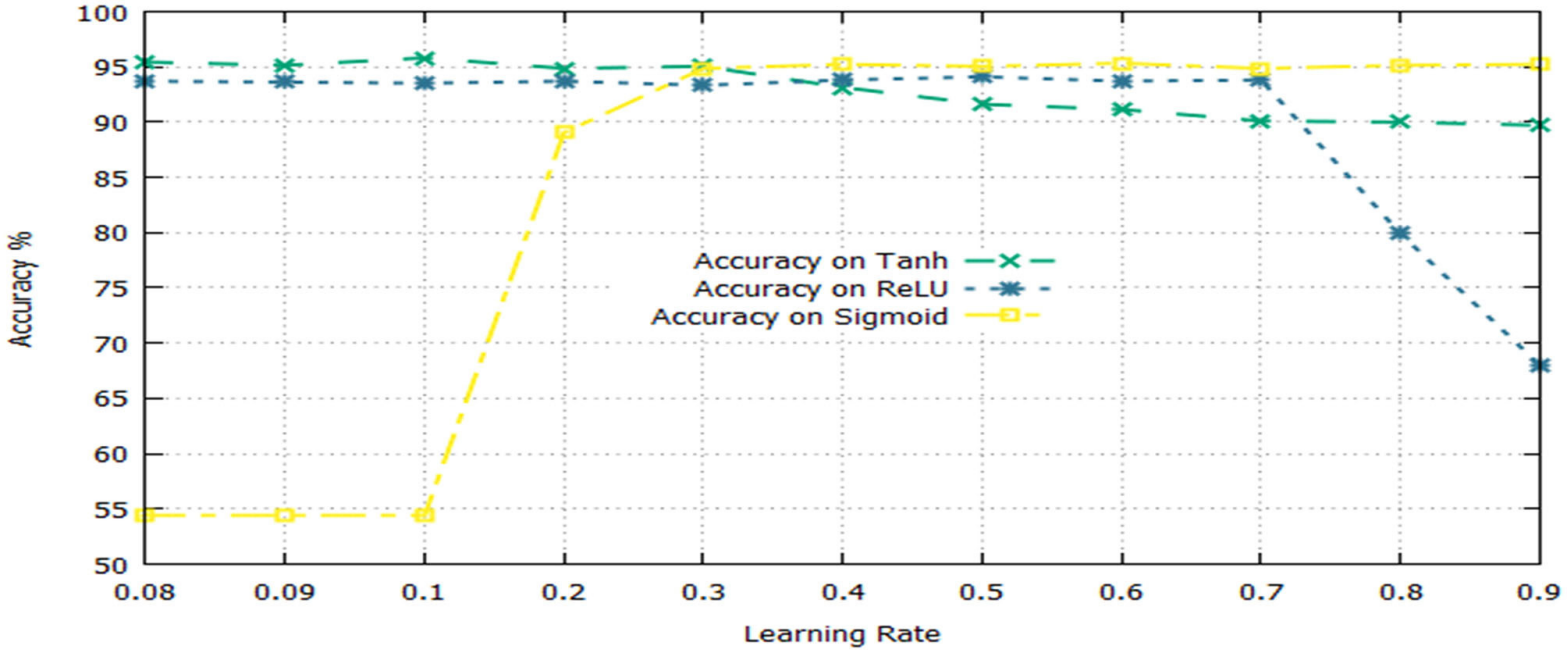
Impact of learning rates and activation function on the performance of DNN.

The activation function is a significant component of a deep learning algorithm. It is a non-linear function employed at a neuron and computes the output of a hidden layer. The activation function decides either a neuron should be fired or ignored based on the information computed at a hidden layer. Different activation functions can be applied in deep learning algorithms, however, the commonly applied activation functions are: sigmoid, Tanh, and Rectified Linear Unit (ReLU). The impact of both the learning rate and activation functions are reported in Table 6 and illustrated in Figure 3.

**Table 6:** Impact of learning rates and activation functions on the accuracy of DNN model.

**Learning rates**	**Tanh (%)**	**ReLU (%)**	**Sigmoid (%)**
0.08	95.43	93.71	54.48
0.09	95.14	93.62	54.48
0.1	95.81	93.52	54.48
0.2	94.86	93.71	89.05
0.3	95.05	93.33	94.86
0.4	93.14	93.81	95.24
0.5	91.62	94.10	95.05
0.6	91.14	93.71	95.33
0.7	90.10	93.81	94.86
0.8	90.00	79.90	95.14
0.9	89.71	68.00	95.24

Table 6 shows that the DNN model achieved a highest accuracy, i.e., 95.81% using Tanh with a combination of learning rate 0.1. The second highest accuracy, i.e., 95.33% reported the sigmoid with a combination of learning rate 0.6. The ReLU yielded the third-highest success rate, i.e., 94.10% with a combination of learning rate 0.5. Furthermore, it can be noted from Figure 3, the un-tune parameter can significantly affect the performance and stability of the model. Moreover, the model shows a stable performance using Tanh compared with other activation functions. Hence, the Tanh can be considered an optimum value for the activation function parameter.

**Figure 4:**
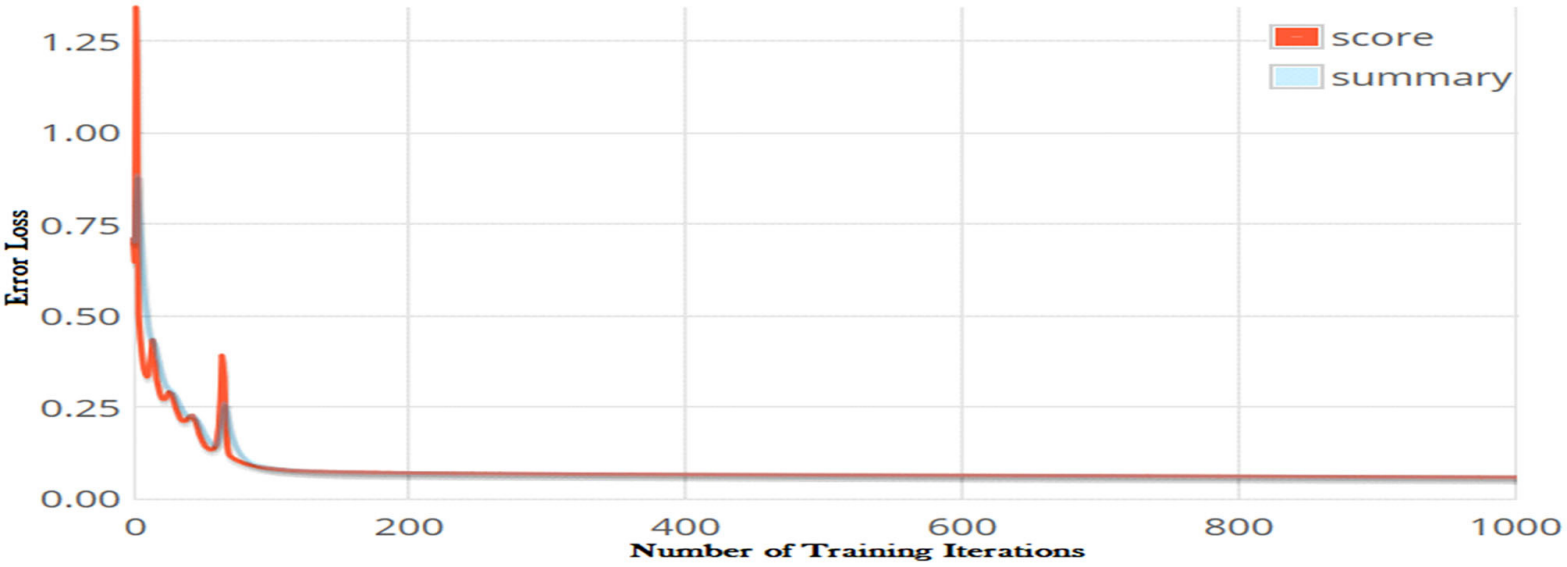
Error losses of DNN model on different iterations using Tanh activation function.

The number of iteration is another optimization parameter and significantly impacts the performance of a learning model. Increasing the number of iterations can significantly minimize the loss function of a model; however, it may increases the model training time considerably. The error loss of the DNN model on different iterations is shown in Figure 4. It can be observed from the figure that increasing the number of iterations significantly reduced loss function. The minimum error loss, i.e., 0.0002176 was reported at iteration 1,000 as shown in the figure. We further increased the number of iterations; however, it did not significantly affect the loss function.

### Performance of DNN Model Using Different Feature Extraction Methods

The performance of the proposed DNN model was analyzed using different feature extraction methods along with the hybrid features, as discussed in section Sequence Formulation Methods. The analysis results are presented in Table 7. From Table 7, we can observe that the DNN model accomplished a highest accuracy of 95.81% using features represented in the G3 group whereas, the lowest accuracy achieved using PseTNC method.

**Table 7:** Performance of DNN Model using different feature extraction methods.

**Groups/Methods**	**SN (%)**	**SP (%)**	**ACC (%)**	**MCC**
Gap	86.68	79.60	82.29	0.6458
Reverse	82.87	80.58	81.52	0.6270
PseTNC	75.78	77.17	76.57	0.5261
G1	94.39	95.78	95.14	0.9021
G2	83.73	85.08	84.48	0.6866
G3	96.17	95.52	95.81	0.9155
G4	86.65	86.17	86.38	0.7250
G5	94.15	95.27	94.76	0.8944
G6	91.18	92.33	91.81	0.8348
G7	93.25	94.08	93.70	0.8729
G8	87.58	86.14	86.76	0.7328
G9	90.85	92.79	91.90	0.8369

**Figure 5:**
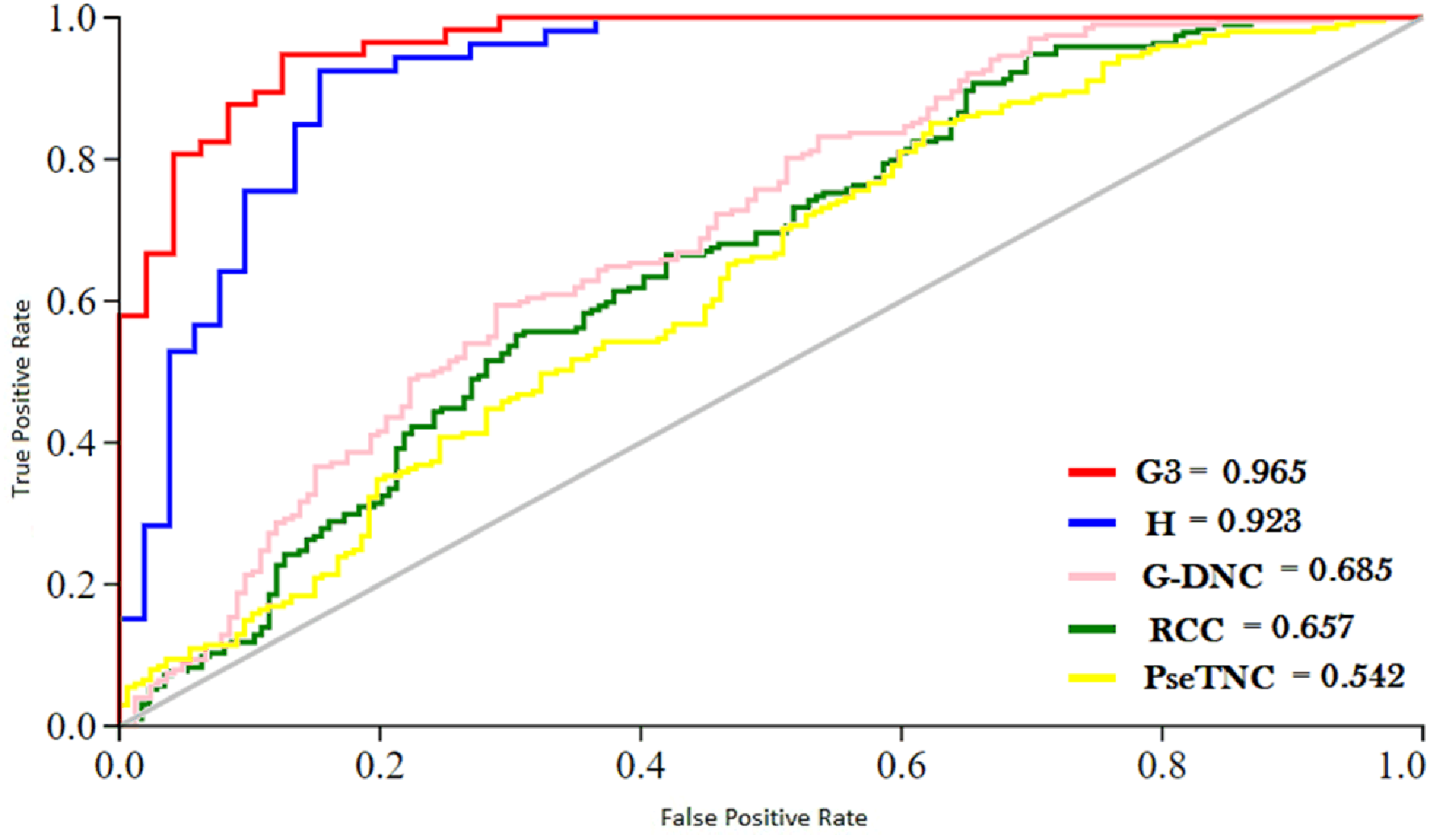
ROC of DNN model using different feature group.

ROC (Receiver Operating characteristic)/AUC (Area Under ROC) curve is another effective method that measure the quality of a prediction model. We evaluated the performance of the DNN model on different feature extraction groups using ROC curve as shown in Figure 5. The figure shows that the DNN model generated a highest value, i.e., 0.965 of ROC/AUC using G3 group features compared with others groups features.

### Comparison of DNN Model With Different Machine Learning Algorithms

**Figure 6:**
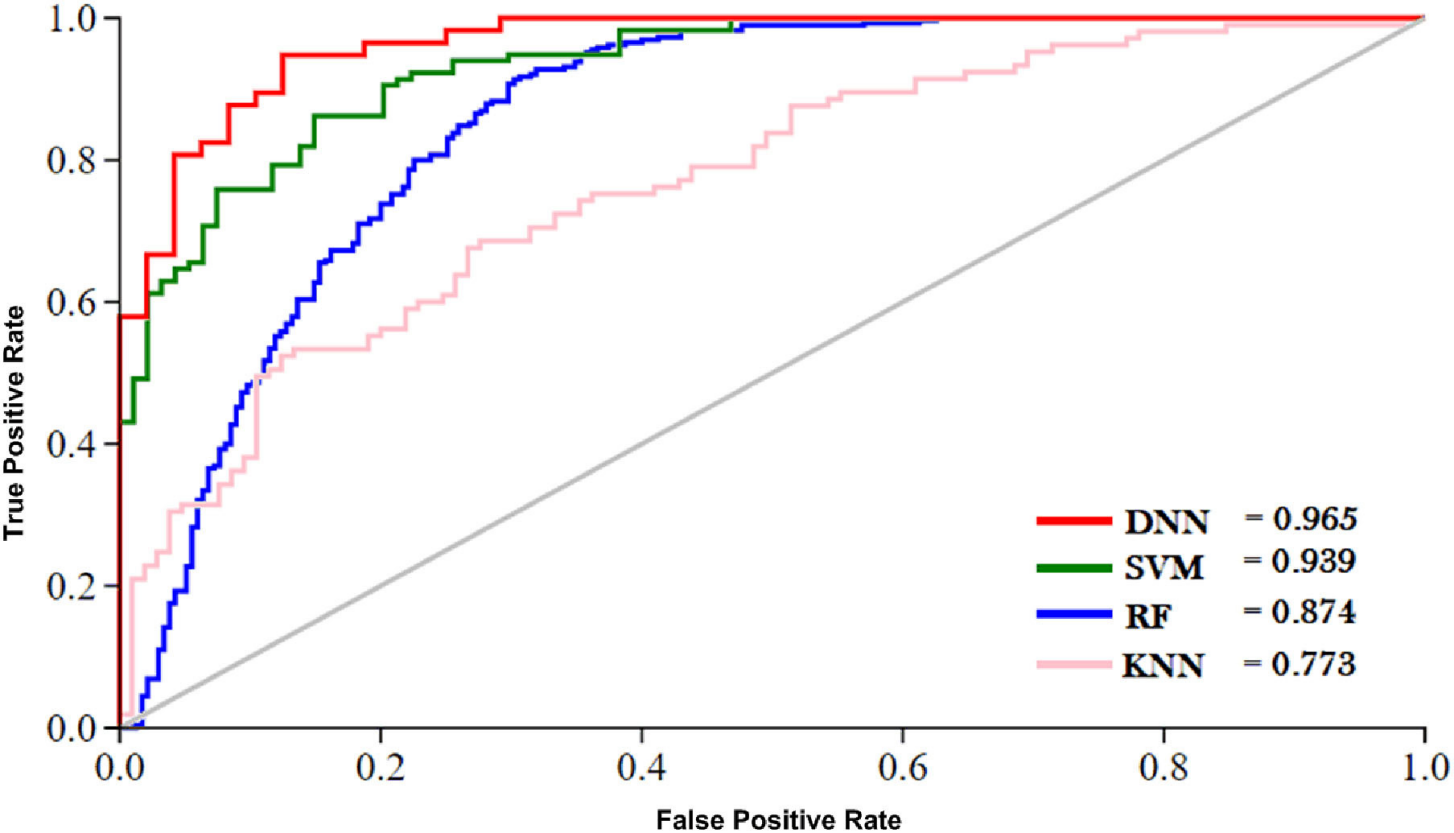
ROC of different classifiers.

The outcome of the DNN in comparison with different learning classifiers, including SVM (Yue et al., 2003), KNN (Cheng et al., 2014), and RF (Fawagreh et al., 2014) is presented in this section. We employed 10-fold cross-validation tests to assess the outcome of the classifiers using different sequence formulation methods. The results of this comparison are shown in Table 8. The table shows that the DNN model achieved a highest accuracy, i.e., 95.81% compared with other machine learning algorithms. The second highest accuracy (i.e., 93.05) reported by the SVM and third-ranking accuracy (i.e., 84.57) obtained by the RF algorithm. The KNN classifier achieved the lowest accuracy (i.e., 75.43). Additionally, we evaluated the performance of the classifiers in more comprehensive way using ROC curve. For the ROC curve, we used only G3 group features for all the classifiers because they generated promising results using G3 group features as shown in Table 8. The ROC curve of the classifiers is shown in Figure 6. From the figure we can observe that the proposed DNN model generated a highest ROC/AUC value, i.e., 0.965 compared with the ROC/AUC values of the other classifiers.

**Table 8:** Performance comparison of DNN with other classifiers.

**Classification algorithm**	**Feature method**	**ACC (%)**	**SN (%)**	**SP (%)**	**MCC**
SVM	Gapped di-nucleotide composition	82.67	72.80	90.91	0.6534
	Reverse complement composition	54.48	–	–	–
	PseTri-nucleotide composition	80.19	70.08	88.64	0.6022
	H	93.05	92.68	93.36	0.860
	G3	92.95	92.68	93.18	0.858
KNN	Gapped di-nucleotide composition	74.67	47.91	94.03	0.5283
	Reverse complement composition	69.90	35.98	95.25	0.4504
	PseTri-nucleotide composition	74.86	48.12	95.20	0.5329
	H	75.24	71.13	78.67	0.499
	G3	75.43	71.34	78.85	0.5035
RF	Gapped di-nucleotide composition	80.19	74.69	84.79	0.5996
	Reverse complement composition	80.29	75.94	83.92	0.6015
	PseTri-nucleotide composition	81.71	75.73	86.71	0.6307
	H	84.57	80.75	87.76	0.688
	G3	84.57	78.87	89.34	0.6888
DNN	Gapped di-nucleotide composition	82.29	86.68	79.60	0.6458
	Reverse complement composition	81.52	82.87	80.58	0.6270
	PseTri-nucleotide composition	76.57	75.78	77.17	0.5261
	H	95.14	94.39	95.52	0.9021
	G3	95.81	96.17	95.78	0.9155

### Comparison of the Proposed Predictor With Existing Predictors

This section presents a performance comparison of the proposed predictor with the existing predictors. For the comparison, we selected 9 recently published predictors from the literature. These predictors are: RF-DYMHC (Jiang et al., 2007b), IDQD (Liu et al., 2012), iRSpot-PseDNC (Chen et al., 2013), iRSpotTNCPseAAC (Qiu et al., 2014), iRSpot-DACC (Liu et al., 2016), iRSpot-EL (Liu et al., 2017a), iRSpot-ADPM (Zhang and Kong, 2018a), iRSpot-SPI (Khan et al., 2019b), and iRecSpot-EF (Jani et al., 2018). For this comparison, the proposed model utilized G3 group features. Results of this comparison in terms of accuracy, sensitivity, specificity, and MMC are listed in Table 9. It is evidently presented in Table 9 that the proposed model outperformed the existing models in terms of all four metrics. For example, consider the MCC metric, the proposed model achieved the highest value, i.e., 0.9155, the second highest value, i.e., 0.9037 reported by the iRecSpot-EF and the third highest value, i.e., 0.8101 generated by the iRSpot-SPI. Similarly, in case of the accuracy metric, the iRecSpot-EF generated 95.14% accuracy whereas, the proposed iRSpot-DNN reported 95.81% accuracy and the iRSpot-SPI produced 90.04% accuracy. These results confirmed that the proposed model outperformed the existing models and can predict the recombination spots with high precision.

**Table 9:** Comparison of the proposed model with existing predictors.

**Methods**	**SN (%)**	**SP (%)**	**MCC**	**ACC (%)**
RF-DYMHC (Jiang et al., 2007b)	73.01	86.56	0.6049	80.40
IDQD (Liu et al., 2012)	79.52	81.82	0.6160	80.77
iRSpot-PseDNC (Chen et al., 2013)	71.75	85.84	0.5830	79.33
iRSpot-TNCPseAAC (Qiu et al., 2014)	76.56	70.99	0.4737	73.52
iRSpot-DACC (Liu et al., 2016)	75.71	88.16	0.6470	82.52
iRSpot-EL (Liu et al., 2017a)	75.29	88.81	0.6510	82.65
iRSpot-ADPM (Zhang and Kong, 2018a)	77.19	90.73	0.6905	84.57
iRSpot-SPI (Khan et al., 2019b)	92.21	92.11	0.8101	90.04
iRecSpot-EF (Jani et al., 2018)	95.14	95.80	0.9037	95.14
Proposed iRSpot-DNN	96.17	95.89	0.9155	95.81

## Conclusion and Future Work

This study presented an intelligent computation model for the identification of recombination spots. In the proposed model, hybrid features were extracted from the benchmark dataset. A novel formula was derived for feature selection and it has 2-fold advantages. Firstly, it avoids biasness among different selected feature extraction algorithms. Secondly, it preserved the sequence discriminant properties along with the sequence-structure information. The performance of the proposed model was investigated on different classifiers. The results exhibit that the optimized deep neural network obtained higher performance having accuracy of 95.81%. As compared to the existing methods the proposed model performance on the prediction of recombination spots is clearly improved. It is realized that the proposed predictor can be considered a handy identification tool and potentially apply to basic research and drug discovery. In future work, we will design a public web server for the proposed iRSpot-DNN so that every experimental scientist can easily access and use for identification of recombination spots.

## Data Availability Statement

The raw data supporting the conclusions of this article will be made available by the authors, without undue reservation, to any qualified researcher.

## Author Contributions

FK, MK, and NI contributed in the main idea of this research, the model concept design, and supervised. FK and SK contributed the code development and conducted experiments. FK wrote the first draft of the manuscript. MK, NI, and DM performed the statistical analysis. MK, NI, AK, and D-QW contributed the results analysis and manuscript revision. All authors contributed to manuscript revision, read, and approved the submitted version.

## Conflict of Interest

The authors declare that the research was conducted in the absence of any commercial or financial relationships that could be construed as a potential conflict of interest.
